# Supplementary material for: Perceptually relevant remapping of human somatotopy in 24 hours
Source: eLife. 2016 Dec 30;5:e17280. doi: 10.7554/eLife.17280 (PMC5241114; doi:10.7554/eLife.17280)
Supplement: Supplementary file 1. — The observed consistency was quantified with Cronbach’s α for each digit pair across the three time points. The resulting values support a high degree of consistency in the relative peak-to-peak distance across the observed digit maps over time. DOI: http://dx.doi.org/10.7554/eLife.17280.015 [file elife-17280-supp1.docx]

|  | **D2-D3 (mm)** | | | **D3-D4 (mm)** | | | **D4-D5 (mm)** | | |
| --- | --- | --- | --- | --- | --- | --- | --- | --- | --- |
|  | C1 | C2 | Glue | C1 | C2 | Glue | C1 | C2 | Glue |
| 1 | 10.99 | 11.68 | 12.41 | 6.66 | 5.18 | 5.64 | 4.25 | 7.40 | 8.99 |
| 2 | 4.62 | 3.19 | 7.24 | 10.64 | 16.21 | 10.11 | 11.25 | 11.09 | 11.83 |
| 3 | 13.64 | 9.72 | 15.78 | 5.65 | 6.40 | 5.34 | 6.54 | 15.81 | 8.13 |
| 4 | 5.45 | 5.14 | 5.45 | 7.35 | 5.19 | 5.34 | 5.51 | 4.67 | 8.04 |
| 5 | 14.83 | 15.88 | 15.31 | 4.04 | 3.89 | 3.73 | 11.33 | 10.43 | 10.43 |
| 6 | 16.42 | 15.42 | 14.45 | 7.73 | 5.81 | 7.73 | 3.73 | 3.00 | 4.04 |
| 6 | 10.32 | 11.03 | 9.52 | 12.10 | 8.63 | 10.38 | 4.85 | 8.00 | 4.05 |
| 8 | 3.02 | 4.08 | 3.88 | 8.93 | 5.46 | 6.18 | 2.66 | 3.97 | 4.13 |
| 9 | 16.65 | 16.49 | 16.65 | 3.20 | 3.24 | 3.00 | 3.93 | 3.82 | 4.38 |
| Cronbach’s  α | 0.975 | | | 0.906 | | | 0.857 | | |

**Supplementary file 1.** *Consistent patterns of peak-to-peak distance (mm) in cortical z-statistic digit representations.* The observed consistency was quantified with Cronbach’s α for each digit pair across the three time points. The resulting values support a high degree of consistency in the relative peak-to-peak distance across the observed digit maps over time.
