# Supplementary material for: Perceptually relevant remapping of human somatotopy in 24 hours
Source: eLife. 2016 Dec 30;5:e17280. doi: 10.7554/eLife.17280 (PMC5241114; doi:10.7554/eLife.17280)
Supplement: Supplementary file 2. — These values support a high degree of consistency in the model fit within each participant and digit across the fMRI time points: 181 TRs of fMRI data. DOI: http://dx.doi.org/10.7554/eLife.17280.016 [file elife-17280-supp2.docx]

|  | **D2 r-value** | | | **D3 r-value** | | | **D4 r-value** | | | **D5 r-value** | | |
| --- | --- | --- | --- | --- | --- | --- | --- | --- | --- | --- | --- | --- |
|  | C1 | C2 | Glue | C1 | C2 | Glue | C1 | C2 | Glue | C1 | C2 | Glue |
| 1 | 0.448 | 0.455 | 0.405 | 0.399 | 0.413 | 0.464 | 0.384 | 0.403 | 0.390 | 0.434 | 0.425 | 0.508 |
| 2 | 0.353 | 0.380 | 0.389 | 0.368 | 0.341 | 0.355 | 0.360 | 0.257 | 0.331 | 0.317 | 0.359 | 0.395 |
| 3 | 0.455 | 0.403 | 0.371 | 0.409 | 0.433 | 0.439 | 0.426 | 0.449 | 0.416 | 0.467 | 0.437 | 0.461 |
| 4 | 0.343 | 0.315 | 0.368 | 0.371 | 0.347 | 0.365 | 0.393 | 0.392 | 0.434 | 0.333 | 0.320 | 0.422 |
| 5 | 0.483 | 0.442 | 0.435 | 0.475 | 0.470 | 0.417 | 0.336 | 0.317 | 0.327 | 0.340 | 0.340 | 0.325 |
| 6 | 0.368 | 0.345 | 0.352 | 0.396 | 0.383 | 0.381 | 0.313 | 0.352 | 0.363 | 0.387 | 0.331 | 0.430 |
| 6 | 0.312 | 0.281 | 0.374 | 0.363 | 0.359 | 0.390 | 0.328 | 0.322 | 0.323 | 0.379 | 0.341 | 0.309 |
| 8 | 0.354 | 0.352 | 0.351 | 0.384 | 0.334 | 0.391 | 0.350 | 0.346 | 0.352 | 0.396 | 0.382 | 0.388 |
| 9 | 0.336 | 0.324 | 0.335 | 0.386 | 0.373 | 0.366 | 0.318 | 0.353 | 0.307 | 0.320 | 0.328 | 0.316 |
| Cronbach’s  α | 0.8868 | | | 0.8705 | | | 0.8789 | | | 0.8542 | | |

**Supplementary file 2.** *Summary of goodness of fit between phase-encoding model and fMRI data for each digit and timepoint.* These values support a high degree of consistency in the model fit within each participant and digit across the fMRI time points: 181 TRs of fMRI data.
