# Supplementary material for: Perceptually relevant remapping of human somatotopy in 24 hours
Source: eLife. 2016 Dec 30;5:e17280. doi: 10.7554/eLife.17280 (PMC5241114; doi:10.7554/eLife.17280)
Supplement: Supplementary file 3. — F: female, M: Male, R: right handed. DOI: http://dx.doi.org/10.7554/eLife.17280.017 [file elife-17280-supp3.docx]

|  | ID | Age | Gender | Handedness | Oldfield Score |
| --- | --- | --- | --- | --- | --- |
| MRI | 1 | 23 | M | R | +80 |
|  | 2 | 23 | F | R | +92 |
|  | 3 | 30 | F | R | +82 |
|  | 4 | 28 | M | R | +94 |
|  | 5 | 33 | M | R | +84 |
|  | 6 | 32 | F | R | +90 |
|  | 7 | 20 | M | R | +88 |
|  | 8 | 20 | F | R | +96 |
|  | 9 | 25 | M | R | +90 |
| Behavioural psychophysics | 10 | 19 | M | R | +100 |
|  | 11 | 20 | M | R | +100 |
|  | 12 | 19 | M | R | +100 |
|  | 13 | 20 | F | R | +66 |
|  | 14 | 19 | F | R | +88 |
|  | 15 | 20 | F | R | +66 |
|  | 16 | 19 | M | R | +100 |
|  | 17 | 19 | F | R | +100 |
|  | 18 | 20 | M | R | +100 |

**Supplementary file 3.** *Demographic information for participants recruited to fMRI and behavioural cohorts.* F: female, M: Male, R: right handed.
