# Supplementary material for: Perceptually relevant remapping of human somatotopy in 24 hours
Source: eLife. 2016 Dec 30;5:e17280. doi: 10.7554/eLife.17280 (PMC5241114; doi:10.7554/eLife.17280)
Supplement: Supplementary file 4. — DOI: http://dx.doi.org/10.7554/eLife.17280.018 [file elife-17280-supp4.docx]

**Supplementary file 4: Statistics overview**

***Inter-digit digit overlap (phase-encoding fMRI)***

*Two-way repeated-measures ANOVA*

Factor 1: Session (Control 1, Control 2, Glued)

Factor 2: Digit pair (D2-D3, D3-D4, D4-D5)

Mauchly’s test of sphericity: W= .215 p= .378

Interaction (Session*Digit pair): F_(4,32)_= 13.412, p<.0005, η^2^= .626

*Post-hoc simple main effects of condition*

*Digit pair: D2-D3*

Mauchly’s test of sphericity: W= .973 p= .910

Main effect of condition: F_(2,16)_= 2.185, p=.145

Pairwise comparisons

| (I) Condition | (J) Condition | Mean Difference (I-J) | Std. Error | Sig.^b^ | 95% Confidence Interval for Difference^a^ | |
| --- | --- | --- | --- | --- | --- | --- |
|  |  |  |  |  | Lower Bound | Upper Bound |
| Control 1 | Control 2 | .006 | .024 | .994 | -.067 | .079 |
|  | Glued | .051 | .027 | .276 | -.032 | .133 |
| Control 2 | Control 1 | -.006 | .024 | .994 | -.079 | .067 |
|  | Glued | .045 | .028 | .373 | -.038 | .128 |
| Glued | Control 1 | -.051 | .027 | .276 | -.133 | .032 |
|  | Control 2 | -.045 | .028 | .373 | -.128 | .038 |
| Pairwise comparisons based on estimated marginal means | | | | | | |
| b. Adjustment for multiple comparisons: Sidak. | | | | | | |

*Digit pair: D3-D4*

Mauchly’s test of sphericity: W= .815 p= .490

Main effect of condition: F_(2,16)_= 23.379, p<.0005

Pairwise comparisons

| (I) Condition | (J) Condition | Mean Difference (I-J) | Std. Error | Sig.^b^ | 95% Confidence Interval for Difference^b^ | |
| --- | --- | --- | --- | --- | --- | --- |
|  |  |  |  |  | Lower Bound | Upper Bound |
| Control 1 | Control 2 | .006 | .007 | .805 | -.015 | .027 |
|  | Glued | .057 | .010 | .001 | .027 | .087 |
| Control 2 | Control 1 | -.006 | .007 | .805 | -.027 | .015 |
|  | Glued | .051 | .010 | .003 | .021 | .081 |
| Glued | Control 1 | -.057 | .010 | .001 | -.087 | -.027 |
|  | Control 2 | -.051 | .010 | .003 | -.081 | -.021 |
| Pairwise comparisons based on estimated marginal means | | | | | | |
| a. Adjustment for multiple comparisons: Sidak. | | | | | | |

*Digit pair: D4-D5*

Mauchly’s test of sphericity: W=.586 p=.360

Main effect of condition: F_(2,16)_= 13.384, p<.0005

Pairwise comparisons

| (I) Condition | (J) Condition | Mean Difference (I-J) | Std. Error | Sig.^b^ | 95% Confidence Interval for Difference^b^ | |
| --- | --- | --- | --- | --- | --- | --- |
|  |  |  |  |  | Lower Bound | Upper Bound |
| Control 1 | Control 2 | .008 | .021 | .973 | -.055 | .072 |
|  | Glued | -.142 | .030 | .004 | -.232 | -.052 |
| Control 2 | Control 1 | -.008 | .021 | .973 | -.072 | .055 |
|  | Glued | -.150 | .043 | .025 | -.280 | -.021 |
| Glued | Control 1 | .142 | .030 | .004 | .052 | .232 |
|  | Control 2 | .150 | .043 | .025 | .021 | .280 |
| Pairwise comparisons based on estimated marginal means | | | | | | |
| a. Adjustment for multiple comparisons: Sidak. | | | | | | |

***Representational similarity analysis (Block design fMRI)***

*Two-way repeated-measures ANOVA*

Factor 1: Session (Control 1, Control 2, Glued)

Factor 2: Digit pair (D2-D3, D3-D4, D4-D5)

Mauchly’s test of sphericity: W= .180 p= .003

Huynh-Feldt correction applied

Interaction (Session*Digit pair): F_(2.0,16.2)_= 4.430, p= .029, η^2^= .356

*Post-hoc simple main effects of condition*

*Digit pair: D2-D3*

Mauchly’s test of sphericity: W= .485 p= .079

Main effect of condition: F_(2,16)_= .328, p= .725

| (I) Condition | (J) Condition | Mean Difference (I-J) | Std. Error | Sig.^b^ | 95% Confidence Interval for Difference^b^ | |
| --- | --- | --- | --- | --- | --- | --- |
|  |  |  |  |  | Lower Bound | Upper Bound |
| Control 1 | Control 2 | -.007 | .033 | .996 | -.105 | .092 |
|  | Glued | -.032 | .054 | .923 | -.195 | .132 |
| Control 2 | Control 1 | .007 | .033 | .996 | -.092 | .105 |
|  | Glued | -.025 | .034 | .854 | -.126 | .076 |
| Glued | Control 1 | .032 | .054 | .923 | -.132 | .195 |
|  | Control 2 | .025 | .034 | .854 | -.076 | .126 |
| Pairwise comparisons based on estimated marginal means | | | | | | |
| a. Adjustment for multiple comparisons: Sidak. | | | | | | |

*Digit pair: D3-D4*

Mauchly’s test of sphericity: W= .580 p= .148

Main effect of condition: F_(2,16)_= 16.076, p= <.0005

| (I) Condition | (J) Condition | Mean Difference (I-J) | Std. Error | Sig.^b^ | 95% Confidence Interval for Difference^b^ | |
| --- | --- | --- | --- | --- | --- | --- |
|  |  |  |  |  | Lower Bound | Upper Bound |
| Control 1 | Control 2 | .013 | .011 | .611 | -.021 | .047 |
|  | Glued | -.084^*^ | .021 | .010 | -.146 | -.022 |
| Control 2 | Control 1 | -.013 | .011 | .611 | -.047 | .021 |
|  | Glued | -.098^*^ | .022 | .007 | -.165 | -.031 |
| Glued | Control 1 | .084^*^ | .021 | .010 | .022 | .146 |
|  | Control 2 | .098^*^ | .022 | .007 | .031 | .165 |
| Pairwise comparisons based on estimated marginal means | | | | | | |
| a. Adjustment for multiple comparisons: Sidak. | | | | | | |

*Digit pair: D4-D5*

Mauchly’s test of sphericity: W= .221 p= .005

Huynh-Feldt correction applied

Main effect of condition: F_(1.180,9.442)_= 7.893 , p= .017

| (I) Condition | (J) Condition | Mean Difference (I-J) | Std. Error | Sig.^b^ | 95% Confidence Interval for Difference^b^ | |
| --- | --- | --- | --- | --- | --- | --- |
|  |  |  |  |  | Lower Bound | Upper Bound |
| Control 1 | Control 2 | -.008 | .006 | .494 | -.025 | .009 |
|  | Glued | .048 | .017 | .068 | -.003 | .099 |
| Control 2 | Control 1 | .008 | .006 | .494 | -.009 | .025 |
|  | Glued | .056 | .019 | .058 | -.002 | .113 |
| Glued | Control 1 | -.048 | .017 | .068 | -.099 | .003 |
|  | Control 2 | -.056 | .019 | .058 | -.113 | .002 |
| Pairwise comparisons based on estimated marginal means | | | | | | |
| a. Adjustment for multiple comparisons: Sidak. | | | | | | |

**Behavioural psychophysics: *Temporal order judgment task***

*Two-way repeated-measures ANOVA*

Factor 1: Session (Control 1, Control 2, Glued)

Factor 2: Digit pair (D2-D3, D3-D4, D4-D5)

Mauchly’s test of sphericity: W= .215 p= .522

Interaction (Session*Digit pair): F_(4,28)_= 14.613, p<.0005 η^2^= .676

*Post-hoc simple main effects of condition*

*Digit pair: D2-D3*

Mauchly’s test of sphericity: W= .833 p= .578

Main effect of condition: F_(2,14)_= 3.456, p= .060

Pairwise comparisons

| (I) Condition | (J) Condition | Mean Difference (I-J) | Std. Error | Sig.^a^ | 95% Confidence Interval for Difference^a^ | |
| --- | --- | --- | --- | --- | --- | --- |
|  |  |  |  |  | Lower Bound | Upper Bound |
| Control 1 | Control 2 | .047 | .018 | .102 | -.009 | .102 |
|  | Glued | .050 | .025 | .238 | -.028 | .128 |
| Control 2 | Control 1 | -.047 | .018 | .102 | -.102 | .009 |
|  | Glued | .003 | .020 | .998 | -.059 | .066 |
| Glued | Control 1 | -.050 | .025 | .238 | -.128 | .028 |
|  | Control 2 | -.003 | .020 | .998 | -.066 | .059 |

Pairwise comparisons based on estimated marginal means

a. Adjustment for multiple comparisons: Sidak.

*Digit pair: D3-D4*

Mauchly’s test of sphericity: W= .688 p= .325

Main effect of condition: F_(2,14)_= 14.631, p<.0005

Pairwise comparisons

| (I) Condition | (J) Condition | Mean Difference (I-J) | Std. Error | Sig.^a^ | 95% Confidence Interval for Difference^b^ | |
| --- | --- | --- | --- | --- | --- | --- |
|  |  |  |  |  | Lower Bound | Upper Bound |
| Control 1 | Control 2 | .006 | .014 | .968 | -.037 | .048 |
|  | Glued | -.075 | .015 | .004 | -.120 | -.029 |
| Control 2 | Control 1 | -.006 | .014 | .968 | -.048 | .037 |
|  | Glued | -.080 | .021 | .018 | -.145 | -.016 |
| Glued | Control 1 | .075 | .015 | .004 | .029 | .120 |
|  | Control 2 | .080 | .021 | .018 | .016 | .145 |

Pairwise comparisons based on estimated marginal means

a. Adjustment for multiple comparisons: Sidak.

*Digit pair: D4-D5*

Mauchly’s test of sphericity: W= .376 p= .246

Main effect of condition: F_(2,14)_= 10.578, p= .002

Pairwise comparisons

| (I) Condition | (J) Condition | Mean Difference (I-J) | Std. Error | Sig.^a^ | 95% Confidence Interval for Difference^b^ | | |
| --- | --- | --- | --- | --- | --- | --- | --- |
|  |  |  |  |  | Lower Bound | Upper Bound | |
| Control 1 | Control 2 | .010 | .006 | .329 | -.008 | .029 | |
|  | Glued | .081 | .025 | .040 | .004 | .157 | |
| Control 2 | Control 1 | -.010 | .006 | .329 | -.029 | .008 | |
|  | Glued | .070 | .021 | .039 | .004 | .136 | |
| Glued | Control 1 | -.081 | .025 | .040 | -.157 | -.004 | |
|  | Control 2 | -.070 | .021 | .039 | -.136 | -.004 | |
| Pairwise comparisons based on estimated marginal means  a. Adjustment for multiple comparisons: Sidak. | | | | | | |  |
|  | | | | | | | |

***Motor confusion task***

*Two-way repeated-measures ANOVA*

Factor 1: Session (Control 1, Control 2, Glued)

Factor 2: Digit pair (D2-D3, D3-D4, D4-D5)

Mauchly’s test of sphericity: W= .068 p= .055

Interaction (Session*Digit pair): F_(4,32)_= 3.828, p= .012 η^2^= .324

*Post-hoc simple main effects of condition*

*Digit pair: D2-D3*

Mauchly’s test of sphericity: W= .876 p= .630

Main effect of condition: F_(2,16)_= 5.550, p= .015 η^2^= .410

Pairwise comparisons

| (I) Condition | (J) Condition | Mean Difference (I-J) | Std. Error | Sig.^a^ | 95% Confidence Interval for Difference^b^ | | |
| --- | --- | --- | --- | --- | --- | --- | --- |
|  |  |  |  |  | Lower Bound | Upper Bound | |
| Control 1 | Control 2 | 7.000 | 3.162 | .163 | -2.501 | 16.501 | |
|  | Glued | -1.667 | 2.315 | .869 | -8.624 | 5.290 | |
| Control 2 | Control 1 | -7.000 | 3.162 | .163 | -16.501 | 2.501 | |
|  | Glued | -8.667^*^ | 2.739 | .039 | -16.895 | -.438 | |
| Glued | Control 1 | 1.667 | 2.315 | .869 | -5.290 | 8.624 | |
|  | Control 2 | 8.667^*^ | 2.739 | .039 | .438 | 16.895 | |
| Pairwise comparisons based on estimated marginal means  a. Adjustment for multiple comparisons: Sidak. | | | | | | |  |

*Digit pair: D3-D4*

Mauchly’s test of sphericity: W= .805 p= .468

Main effect of condition: F_(2,16)_= 1.320, p= .295 η^2^= .142

Pairwise comparisons

| (I) Condition | (J) Condition | Mean Difference (I-J) | Std. Error | Sig.^a^ | 95% Confidence Interval for Difference^b^ | | |
| --- | --- | --- | --- | --- | --- | --- | --- |
|  |  |  |  |  | Lower Bound | Upper Bound | |
| Control 1 | Control 2 | 3.222 | 3.382 | .748 | -6.939 | 13.384 | |
|  | Glued | -2.778 | 4.431 | .908 | -16.091 | 10.535 | |
| Control 2 | Control 1 | -3.222 | 3.382 | .748 | -13.384 | 6.939 | |
|  | Glued | -6.000 | 3.149 | .254 | -15.462 | 3.462 | |
| Glued | Control 1 | 2.778 | 4.431 | .908 | -10.535 | 16.091 | |
|  | Control 2 | 6.000 | 3.149 | .254 | -3.462 | 15.462 | |
| Pairwise comparisons based on estimated marginal means  a. Adjustment for multiple comparisons: Sidak. | | | | | | |  |

*Digit pair: D4-D5*

Mauchly’s test of sphericity: W= .109 p< .005

Huynh-Feldt correction applied

Main effect of condition: F_(2,8.661)_= 10.362, p= .010 η^2^= .564

Pairwise comparisons

| (I) Condition | (J) Condition | Mean Difference (I-J) | Std. Error | Sig.^a^ | 95% Confidence Interval for Difference^b^ | | |
| --- | --- | --- | --- | --- | --- | --- | --- |
|  |  |  |  |  | Lower Bound | Upper Bound | |
| Control 1 | Control 2 | -1.444 | 1.107 | .540 | -4.770 | 1.881 | |
|  | Glued | -18.444^*^ | 5.313 | .025 | -34.407 | -2.482 | |
| Control 2 | Control 1 | 1.444 | 1.107 | .540 | -1.881 | 4.770 | |
|  | Glued | -17.000^*^ | 5.610 | .048 | -33.856 | -.144 | |
| Glued | Control 1 | 18.444^*^ | 5.313 | .025 | 2.482 | 34.407 | |
|  | Control 2 | 17.000^*^ | 5.610 | .048 | .144 | 33.856 | |
| Pairwise comparisons based on estimated marginal means  a. Adjustment for multiple comparisons: Sidak. | | | | | | |  |
